# Protective Effects of Whey Protein Hydrolysate, Treadmill Exercise, and Their Combination against Scopolamine-Induced Cognitive Deficit in Mice

**DOI:** 10.3390/foods12244428

**Published:** 2023-12-10

**Authors:** Yeok Boo Chang, Eun-Jin Jung, Hyung Joo Suh, Hyeon-Son Choi

**Affiliations:** 1 Department of Integrated Biomedical and Life Science, Graduate School, Korea University, Seoul 02841, Republic of Korea; oobkoey@gmail.com; 2 Transdisciplinary Major in Learning Health Systems, Graduate School, Korea University, Seoul 02841, Republic of Korea; 3 Department of Food and Biotechnology, Korea University, Sejong 30019, Republic of Korea; ejjung1124@korea.ac.kr; 4 Department of Food Nutrition, Sangmyung University, Seoul 03016, Republic of Korea

**Keywords:** whey protein hydrolysate, neuroprotective effect, treadmill exercise, scopolamine

## Abstract

In this study, the potential of whey protein hydrolysate (WPH) and treadmill exercise to prevent cognitive decline was investigated, along with their neuroprotective mechanisms. Cognitive dysfunction was induced in mice with 1 mg/kg of scopolamine, followed by the administration of WPH at 100 and 200 mg/kg and/or treadmill exercise at 15 m/min for 30 min five days per week. Both WPH administration and treadmill exercise significantly improved the memory of mice with scopolamine-induced cognitive impairment, which was attributed to several key mechanisms, including a reduction in oxidative stress based on decreased levels of reactive oxygen species and malondialdehyde in the brain tissue and an increase in acetylcholine by increasing choline acyltransferase and decreasing acetylcholine esterase levels. Exercise and WPH also exerted neuroprotective effects by inhibiting the hyperphosphorylation of tau proteins, enhancing the expression of the brain-derived neurotrophic factor, and inhibiting apoptosis by reducing the Bax/Bcl2 ratio in conjunction with the downregulation of the mitogen-activated protein kinase pathway. Moreover, the impact of WPH and treadmill exercise extended to the gut microbiome, suggesting a potential link with cognitive improvement. These findings suggest that both WPH intake and treadmill exercise are effective strategies for mitigating cognitive impairment, providing promising avenues for treating neurodegenerative diseases.

## 1. Introduction

Dementia can be broadly classified into Alzheimer’s disease (AD) and vascular dementia based on its mechanism of occurrence. AD is the most prevalent and rapidly progressive type of dementia, mainly affecting individuals over 60 years of age, accounting for 50–60% of all dementia cases [1]. AD can be further subdivided into subtypes triggered by environmental and physiological factors. External factors, including age, genetic determinants, and environmental factors, can lead to toxic effects, whereas internal factors involve a decline in neurotransmitters due to reduced acetylcholine (ACh) levels, neuroinflammation initiated by cytokines, cytotoxicity due to the aggregation of beta-amyloid (Aβ) protein and hyperphosphorylated tau protein, and neuronal demise induced by oxidative stress-generated free radicals. The accumulation of Aβ, an aberrant protein within nerve cells, in the brain results in synaptic damage and the activation of peripheral glial cells, ultimately culminating in brain cell death mediated by various inflammatory, apoptotic, and oxidative stress responses. ACh is a neurotransmitter required for cognitive functions such as learning and memory [2]. Acetylcholinesterase (AChE) inhibitors serve as therapeutic agents that alleviate the symptoms of cognitive decline in dementia by enhancing cholinergic neurotransmitters [3]. Recent studies have investigated the potential inhibitory effects of substances derived from natural products and food materials against ACh-degrading enzymes. Scopolamine, a muscarinic cholinergic receptor antagonist, inhibits cholinergic neuronal function in the central nervous system, leading to oxidative stress and subsequent cognitive impairment [4], offering a useful drug to stimulate cholinergic memory loss in animal models, thereby facilitating studies on the efficacy of various drugs and natural compounds on protecting memory and cognitive functions [5]. 

Exercise has been shown to have a positive effect on neurodegenerative diseases through the release of brain-derived neurotrophic factor (BDNF), the inhibition of neuronal cell death, and promotion of neurogenesis, collectively enhancing cognitive functions related to memory and learning [6,7,8]. Among various exercises, treadmill exercise has been shown to enhance mitochondrial function and promote autophagy, contributing to the breakdown of abnormal proteins and the maintenance of cellular homeostasis, ultimately fostering positive effects on learning and memory [9,10]. In particular, one study indicated that 4 weeks of endurance exercise had a positive impact on the adaptation of the skeletal muscles to physical activity, thereby increasing the expression of proteins involved in mitochondrial biosynthesis and mitophagy in mice [11].

Whey protein is a valuable by-product of the cheese-making process, accounting for approximately 20% of total milk proteins. Whey protein is quickly absorbed by the body and contains a large number of branched-chain amino acids that are widely distributed in the muscles [12]. Enzymatic hydrolysis is a common method utilized to improve the functional and nutritional properties of proteins [13,14]. The peptides produced during protein hydrolysis have smaller molecular weights and altered secondary structures compared to those of intact proteins, with the potential to enhance physiological functions, including digestive ability, and to mitigate allergic reactions [15]. Whey protein hydrolysate (WPH) has been demonstrated to have a range of beneficial effects, including increasing the body’s metabolic rate, promoting intestinal health, regulating blood sugar levels, and managing blood pressure. A recent study demonstrated that WPH containing a glycine–threonine–tryptophan–tyrosine peptide improved cognitive function in mice [16]. Nevertheless, there is a lack of research on the cognitive function improvement resulting from the combination of peptides derived from WPH and aerobic exercise.

To address this question, in this study, we evaluated the individual and combined effects of the administration of WPH and treadmill exercise on cognitive function in mice with scopolamine-induced cognitive decline and further explored the underlying mechanism. 

## 2. Materials and Methods

### 2.1. Preparation of Whey Protein Hydrolysates

Acid whey or whey protein concentrate (WPC) was dissolved in distilled water (50~55 °C) to reach a 20% concentration (*w*/*w*). The solution was adjusted to pH 7 to 7.5 using sodium bicarbonate. Afterwards, 0.2% Alcalase 2.4 L FG (Novozyme, Denmark) and 0.2% Protamex (Novozyme, Denmark) were added to the solution and incubated for 4 h at 50~55 °C. Then, 0.2% Flavourzyme 1000 L (Novozyme, Denmark) was added and incubated for 15 h at 50~55 °C. After the enzyme reactions, the solution was inactivated via boiling at 90 °C for 10 min. The inactivated solution was filtered using a 1 µm filter paper to remove unhydrolyzed WPC or acid whey. Then, they were heat sterilized, dried using a spray dryer, and stored at −20 °C until use.

### 2.2. Experimental Animals and Design

Six-week-old male specific pathogen-free C57BL6 mice weighing 20–25 g were purchased from OrientBio (Seongnam, Republic of Korea). The animals were housed in an environment with a temperature of 23 ± 3 °C, a relative humidity of 50 ± 10%, a ventilation frequency of 10–15 times/h, a 12 h light/dark cycle (08:00 to 20:00), and an illuminance of 150–300 Lux. During the 1-week acclimatization period, the animals were allowed to consume solid laboratory-grade food (Cargil Agri Purina, Inc., Seongnam, Republic of Korea) and drinking water ad libitum. After the acclimatization period, the mice were divided into seven experimental groups with eight mice per group: the normal (NOR), scopolamine control (CON), scopolamine + exercise (EXR), scopolamine + WPH (WPH_L, WPH_H), and scopolamine + exercise + WPH groups (EWPH_L, EWPH_H). The mice in the WPH groups were orally administered 100 (WPH_L/EWPH_L) or 200 mg/kg (WPH_H/EWPH_H) of WPH per day based on their body weight. The mice in the exercise groups were subjected to treadmill exercise five times per week for 30 min at a speed of 15 m/min during the four-week experimental period. To induce cognitive impairment, 1 mg/kg of scopolamine (Sigma-Aldrich Co., St. Louis, MO, USA) was administered intraperitoneally 30 min before the cognitive tests. 

All animal experiments were approved by the Institutional Animal Care and Use Committee of Korea University (KIACUC-2022-0076).

### 2.3. Y-Maze Test

The Y-maze test is a behavioral test commonly used to assess short-term spatial cognition [17]. This assessment involves the use of a Y-shaped maze constructed from a white plastic material. The maze used in this study comprised three arms, each measuring 50 cm in length, 20 cm in height, and 10 cm in width. These arms were folded at an angle of 120° to each other. After designating each branch as A, B, or C, the mouse was placed at the beginning of the maze and allowed to move freely through the maze for 60 s. The number and order of entries into each part of the maze were measured to evaluate the change in behavior. The scoring system for this assessment involved awarding one point when the mouse sequentially entered all three main areas (A, B, and C) in the correct order. However, no points were awarded if entries occurred in a non-sequential manner. The test conductor established the basic conditions for learning and memory assessment criteria and validated the test method in terms of rationality, accuracy, and reproducibility.

### 2.4. Novel Object Recognition Test

The novel object recognition test was performed in a white polyvinyl plastic box (30 cm × 30 cm× 30 cm). The mice were allowed to explore the box freely for 10 min for the first 2 days for acclimation [18]. On the third day of the experiment, the mice were injected with WPH at 100 or 200 mg/kg 1 h before being placed in the box. All experimental groups except for the NOR group were administered an intraperitoneal dose of scopolamine (1 mg/kg) dissolved in a 0.9% saline solution 30 min before the start of the experiment, placed in the front center of the box, and then presented with identical objects at equal distances (5 cm) from both diagonal vertices of the box and allowed to explore the objects freely for 5 min. After 24 h of exploration, one of the objects in the box was replaced with a new object of a different shape and the mouse was allowed to explore the object freely for another 5 min. During these 5 min, we measured the time that the mouse exhibited exploratory behaviors such as touching, sniffing, and licking familiar and novel objects. The object preference ratio and discrimination indices were then calculated by recording the number of touches between the familiar object and the novel object and comparing the total number of touches of the objects. A higher discrimination index indicates a greater recognition of novel objects.

### 2.5. Measurement of ACh Content 

The ACh content in the brain tissue was measured using the modified method of Vincent and Newsom-Davis [19]. In brief, the harvested brain tissue was homogenized in 1 mL of phosphate-buffered saline (PBS), centrifuged (10,000× *g*, 10 min), and the supernatant was obtained for analysis. An alkaline hydroxylamine reagent (3.5 N sodium hydroxide and 2 M hydroxylamine in HCl) was added to the supernatant and left to react at room temperature for 1 min, followed by the addition of 0.1 N HCl in 0.5 N HCl (pH 1.2) and 0.37 M FeCl_3_. The absorbance was then measured at a wavelength of 540 nm.

### 2.6. Measurement of AChE Activity

The AChE activity was measured using an AChE activity assay kit (BM-ACH-100; BIOMAX Co., Ltd., Guri, Republic of Korea) according to the manufacturer’s instructions. The brain tissue was homogenized in 1 mL of PBS containing 1% Triton X-100 (Sigma Aldrich) and centrifuged (12,000× *g* 10 min) to obtain the supernatant. Subsequently, 50 μL of the supernatant was mixed with the reaction mixture containing an assay buffer, enzyme mix, substrate solution, and the probe, and the absorbance was measured at 570 nm with a microplate reader in kinetic mode at 37 °C for 30 min in the dark.

### 2.7. Determination of Reactive Oxygen Species (ROS) and Malondialdehyde (MDA) Contents

To measure ROS levels, 1 mL of 40 mM Tris–HCl buffer was added to 50 mg of brain tissue. The tissues were homogenized and centrifuged to collect the supernatant. Subsequently, 500 μL of 40 mM Tris–HCl buffer and 10 μM of the fluorescent probe 2′,7′-dichlorofluorescein diacetate (DCF-DA) were added to 50 μL of the supernatant and reacted for 30 min at 37 °C. Fluorescence was measured at an excitation wavelength of 482 nm and an emission wavelength of 535 nm to quantify the ROS content in the tissue through a comparison with an ROS standard curve.

The MDA content in the brain tissue was analyzed using the Oxitec^TM^ TBARS Assay kit (BIOMAX Co., Ltd., Guri, Republic of Korea) following the manufacturer’s protocol. Briefly, 1 mL of PBS was added to 100 mg of brain tissue, which was homogenized and centrifuged to collect the supernatant. Subsequently, 200 μL of the indicator solution was added to 200 μL of the supernatant and reacted at 65 °C for 45 min. The absorbance was measured at 450 nm and compared to the MDA standard curve to quantify the MDA content in the tissue.

### 2.8. Western Blotting

Approximately 50 mg of brain tissue was homogenized in 1000 μL of a lysis buffer (200 mM Tris (pH 8.0), 150 mM NaCl, 2 mM EDTA, 1 mM NaF, 1% NP40, 1 mM phenylmethanesulfonyl fluoride, 1 mM Na_3_VO_4_, and a protease inhibitor cocktail) and centrifuged at 12,000× *g* for 5 min at 4 °C to recover the supernatant.

The sample was subjected to protein quantification via the bicinchoninic acid method, and 30 μg of protein was subjected to electrophoresis on a 10% sodium dodecyl sulfate–polyacrylamide gel electrophoresis gel. After transfer to a polyvinylidene fluoride membrane, the blots were blocked with 5% skim milk and bovine serum albumin for 1 h and probed with the following primary antibodies for 16 h at 4 °C: alpha-tubulin (Cell Signaling Technology, Inc., Beverly, MA, USA, Cat. #2144), Bax (Cell Signaling Technology, Inc., Beverly, MA, USA, Cat. #2772), Bcl-2 (Cell Signaling Technology, Inc., Cat. #2876), PARP (Cell Signaling Technology, Inc., Cat. #9553), BDNF (Cell Signaling Technology, Inc., Cat. #47808), phosphor (p)-tau (Cell Signaling Technology, Inc., Cat. #29957), tau (Cell Signaling Technology, Inc., Cat. #4019), and choline acyltransferase (ChAT; abcam, ab183591) antibodies. After washing three times with Tris-buffered saline with Tween 20, the blot was treated with a horseradish peroxidase-conjugated anti-rabbit IgG secondary antibody (Cell Signaling Technology, Inc., Cat. #7074) and reacted for 2 h at room temperature, followed by dispensing the SuperSignal™ Western Blot Enhancer (Thermo Fisher Scientific, Waltham, MA, USA, Cat. #46641), and protein bands were identified using the FluorChem M Fluorescent Western Imaging System (Protein Simple, San Jose, CA, USA). The antibodies used were diluted in 5% skim milk and bovine serum albumin, according to the manufacturer’s instructions.

### 2.9. Gut Microbiome Analysis

DNA extraction and 16S rRNA gene sequencing of double-stranded DNA were performed from 100 mg of cecum samples using the QIAamp Power Fecal Pro DNA Kit (QIAGEN, Frederick, MD, USA) following the manufacturer’s protocol. The DNA concentration of all samples was adjusted to 5 ng/µL, and the uniformly concentrated DNA was subjected to a two-step polymerase chain reaction using the primer set 341F and 806R to amplify the V3–V4 variable region of the 16S rRNA gene. Illumina MiSeq (Illumina, CA, USA) library construction was performed according to the manufacturer’s protocol and sequencing was performed by Macrogen (Seoul, Republic of Korea).

### 2.10. Statistical Analysis

The SPSS software (version 12.0; SPSS Inc., Chicago, IL, USA) was used to analyze the experimental data. The data of each experiment are expressed as percentages or mean ± standard error of the mean as appropriate, and all measurements were subjected to a one-way analysis of variance followed by a post hoc Tukey test to evaluate significance. Statistical significance was judged at a threshold of *p* < 0.05.

## 3. Results

### 3.1. Effect of Treadmill Exercise and WPH on Scopolamine-Induced Cognitive Decline 

**Figure 1:**
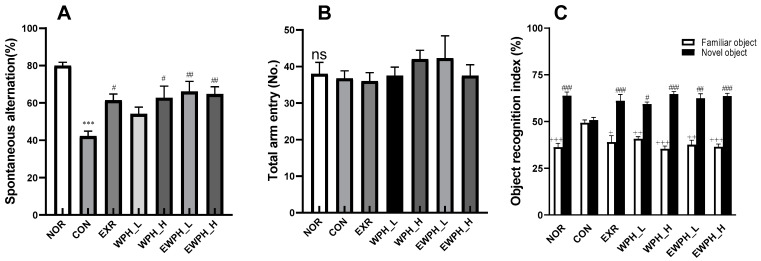
Effect of WPH and treadmill exercise on cognitive behaviors in mice in the Y-maze and novel object recognition tests. Spontaenous aleternation (**A**), Total arm entry (**B**), Novel/Familiar object recognition index (**C**). NOR, normal; CON, control; EXR, exercise; WPH-L/H, low-dose/high-dose whey protein hydrolysate; EWPH_L/H, exercise + low-dose/high-dose whey protein hydrolysate. Data are presented as mean ± standard error of the mean. *** *p* < 0.001 vs. NOR group; ^#^ *p* < 0.05, ^##^ *p* < 0.01, ^###^ *p* < 0.001 vs. CON group; ns, not significant (*p* > 0.05) (analysis of variance followed by post hoc Tukey’s test), ^*+*^ *p* < 0.05, *^++^* *p* < 0.01, and *^+++^* *p* < 0.001 vs. CON groups (Student’s *t*-test).

Y-maze and novel object recognition tests were used to measure the memory improvement conferred by WPH administration and treadmill exercise in mice with scopolamine-induced cognitive impairment (Figure 1). In the Y-maze, the spontaneous alternation in the NOR group (no treatment) was 79.99%, which was higher than that of the CON group (group administered scopolamine and saline) of 42.25% (Figure 1A). The spontaneous alternation increased to 47% in EXR (treadmill exercise). The frequency of spontaneous alternation was increased by 54.1% and 62.7% in a concentration-dependent manner following the administration of 100 and 200 mg/kg of WPH, respectively. The EWPH_L (treadmill exercise with low-concentration WPH administration) and EWPH_H (treadmill exercise with high-concentration WPH administration) groups showed a synergistic effect by increasing the spontaneous alternation by 66.1%, and 64.7%, respectively (Figure 1A). The total number of entries into each arm did not show significant differences among any of the experimental groups (Figure 1B), even though the spontaneous alternations tended to increase when WPH was combined with exercise.

In the novel object recognition test, which evaluates the ability of mice to explore novelty, the discrimination index (Figure 1C) was calculated by analyzing the time taken to explore new and existing objects. The discrimination index for novel objects in the CON group was significantly reduced compared to that of the NOR group (*p* < 0.05), indicating that scopolamine induced a cognitive impairment associated with new object recognition. The discrimination index for novel objects significantly increased in the EXR, WPH_L, WPH_H, EWPH_L, and EWPH_H groups compared with that of the CON group (Figure 1C). Unlike the Y-maze test, the combination of exercise and WPH did not show synergistic effect in the novel recognition test. 

By contrast, the discrimination index for familiar objects significantly increased in the CON group compared to that of the NOR group, but decreased to the level of the NOR group in the EXR, WPH_L, WPH_H, EWPH_L, and EWPH_H groups compared to the CON group. Unlike the NOR, EXR, and WPH groups, the CON group showed similar indices for familiar and novel objects (Figure 1C). This result indicated that both exercise and WPH administration improved the ability of mice to explore novel objects (*p* < 0.001, Figure 1C). No synergistic effect was observed with the combination of treadmill exercise and WPH.

### 3.2. Effects of Treadmill Exercise and WPH on ROS and MDA Levels in Scopolamine-Administered Mice

**Figure 2:**
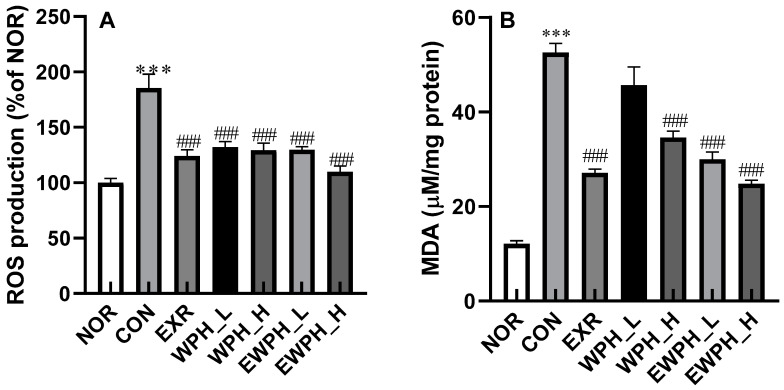
Effect of WPH and treadmill exercise on reactive oxygen species (ROS) and malondialdehyde (MDA) production. ROS level (**A**), malondialdehyde (MDA) level (**B**). NOR, normal; CON, control; EXR, exercise; WPH-L/H, low-dose/high-dose whey protein hydrolysate; EWPH_L/H, exercise ^+^ low-dose/high-dose whey protein hydrolysate. The levels of ROS (**A**) and MDA (**B**) were determined using DCFH-DA and TBRARS assays, respectively. Data are presented as mean ± standard error of the mean. *** *p* < 0.001 vs. NOR group; ^###^ *p* < 0.001 vs. CON group (analysis of variance followed by post hoc Tukey’s test).

The ROS content in the CON group was significantly increased by 86.9% compared to that in the NOR group, indicating that scopolamine induced an increase in ROS production. The administration of 100 and 200 mg/kg of WPH significantly reduced ROS levels by 28.4% and 30.2%, respectively, compared to those of the CON group (Figure 2A). In the EXR group, which only performed treadmill exercise, and the EWPH_L and EWPH_H groups, which combined WPH administration and exercise, the ROS levels were significantly reduced by 32.1%, 30.4%, and 41.2%, respectively, compared to those in the CON group (*p* < 0.001, Figure 2B). These results show that treadmill exercise, WPH, and their combinational administrations inhibited the scopolamine-induced increase in ROS levels responsible for oxidative stress.

The MDA content of the CON group was 52.6 μM, which was significantly higher than that of the NOR group at 12.1 μM (*p* < 0.001). The MDA content of the EXR and WPH_H groups was significantly reduced to 27.1 and 34.6 μM, respectively, compared to that of the CON group (*p* < 0.001, Figure 2B). The MDA content of the groups that combined exercise and WPH administration (EWPH_L and EWPH_H) was 29.9 and 24.8 μM, which was significantly decreased compared to that of the CON group (*p* < 0.001, Figure 2B). This result showed that exercise and WPH administration effectively reduced the scopolamine-induced formation of MDA in the mouse brain.

### 3.3. Effects of Treadmill Exercise and WPH on ChAT Protein Abundance, ACh Content, and AChE Activity in Scopolamine-Administered Mice 

**Figure 3:**
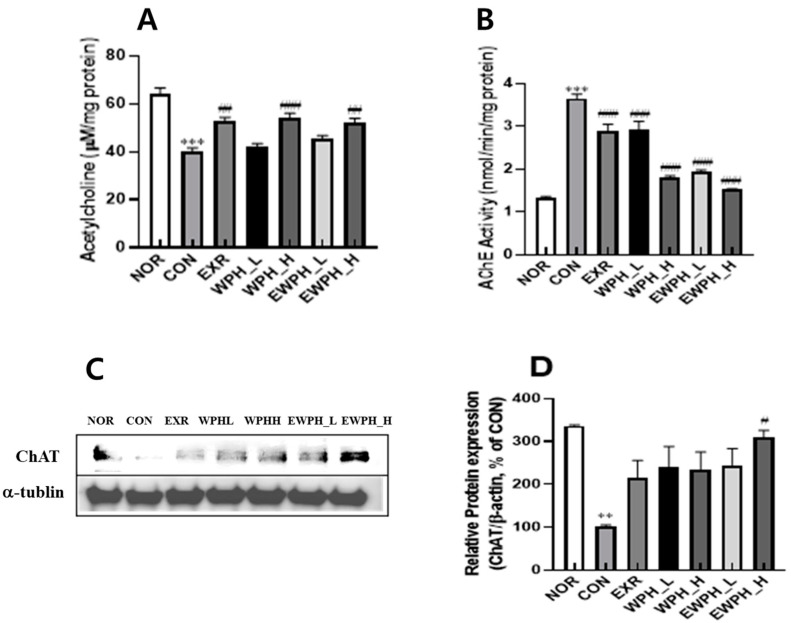
Effect of WPH and treadmill exercise on ACh, AChE, and ChAT. NOR, normal; CON, control; EXR, exercise; WPH-L/H, low-dose/high-dose whey protein hydrolysate; EWPH_L/H, exercise + low-dose/high-dose whey protein hydrolysate; ACh, acetylcholine; AChE, acetylcholine esterase; ChAT, choline acetyltransferase. The ACh content in the brain was measured using a colorimetric assay at 540 nm (**A**). AChE activity was examined using a specific assay kit (**B**). ChAT protein levels from the brain were determined using Western blot (**C**) and quantified using ImageJ (Version 1.53t) (**D**). Data are presented as mean ± standard error of the mean. ** *p* < 0.01, *** *p* < 0.001 vs. NOR group; ^#^ *p* < 0.05, ^##^ *p* < 0.01, ^###^ *p* < 0.001 vs. CON group (analysis of variance followed by post hoc Tukey’s test).

In the mouse brain tissue, the ACh concentration of the CON group was significantly reduced by 0.6 times compared to that of the NOR group (Figure 3A). In addition, the activity of AChE, an ACh-degrading enzyme, was significantly increased by 2.7 times, and the protein level of ChAT was significantly decreased by 3.3 times in the NOR group compared with that of the CON group (Figure 3). These results indicate that the administration of scopolamine reduced ACh levels by increasing AChE activity and decreasing the ChAT protein levels, consequently leading to cognitive dysfunction. Treadmill exercise and WPH administration restored the ACh levels that were reduced by the scopolamine administration (Figure 3A). The ACh concentration significantly increased by 1.3 times in the EXR, WPH_H, and EWPH_H groups compared to that in the CON group (*p* < 0.01, Figure 3A). The AChE activity significantly decreased by 0.8-fold, 0.8-fold, 0.5-fold, 0.5-fold, and 0.4-fold in the EXR, WPH_L, WPH_H, EWPH_L, and EWPH_H groups, respectively (*p* < 0.001; Figure 3B). The expression level of ChAT protein was significantly increased by 309.12% in the EWPH_H group compared to that in the CON group (*p* < 0.05; Figure 3C). The combination of treadmill exercise and a high-dose WPH administration synergistically increased the ChAT protein levels. These results show that both exercise and WPH administration effectively restored ACh levels, which are essential for neurotransmission and cognitive function [20].

### 3.4. Effects of Treadmill Exercise and WPH on Neuronal Apoptosis and Tau Phosphorylation in Scopolamine-Administered Mice

**Figure 4:**
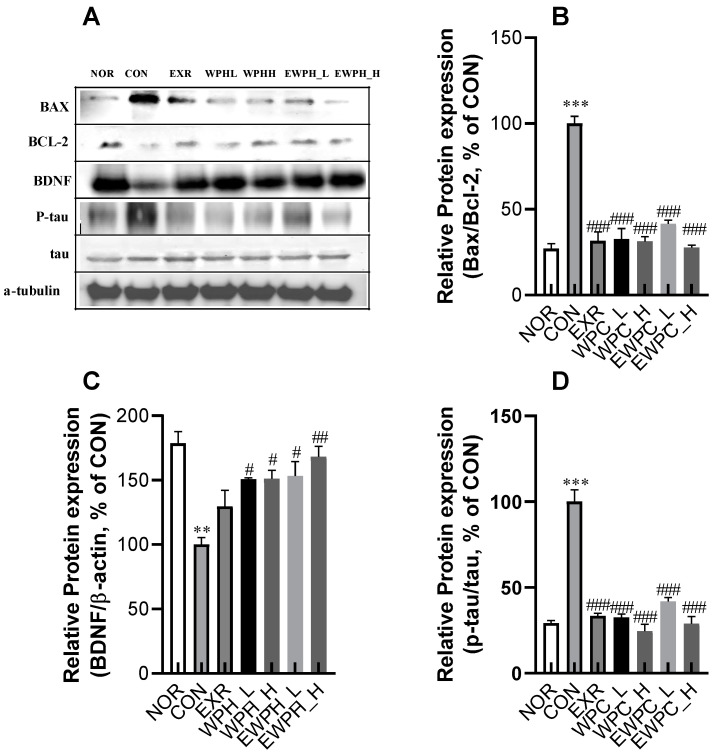
Effect of WPH and treadmill exercise on the relative protein ratios of Bax/Bcl-2, BDNF, and p-tau/tau. NOR, normal; CON, control; EXR, exercise; WPH-L/H, low-dose/high-dose whey protein hydrolysate; EWPH_L/H, exercise + low-dose/high-dose whey protein hydrolysate. Bax, Bcl-2, BDNF, and p-tau, and tau protein levels were determined using Western blot (**A**), and the Bax/Bcl2 (**B**), BDNF (**C**), and p-tau/tau (**D**) ratios were measured using ImageJ. Data are presented as mean ± standard error of the mean. ** *p* < 0.01, *** *p* < 0.001 vs. NOR group; ^#^ *p* < 0.05, ^##^ *p* < 0.01, ^###^ *p* < 0.001 vs. CON group (analysis of variance followed by post hoc Tukey’s test).

The Bax/Bcl-2 ratio was significantly increased in the CON group following scopolamine treatment compared to that in the NOR group (Figure 4A,B). However, the Bax/Bcl-2 ratio significantly decreased in the EXR group subjected to treadmill exercise; the WPH_L and WPH_H groups treated with WPH at low and high doses, respectively; and the EWPH_L and EWPH_H groups receiving both exercise and WPH at low and high doses, respectively (31.6%, 32.7%, 31.3%, 41.6%, and 27.6%, respectively; *p* < 0.001, Figure 4B). Therefore, exercise and WPH administration effectively inhibited scopolamine-induced apoptosis by reducing the Bax/Bcl-2 ratio. These results demonstrate that both exercise and WPH administration effectively inhibited apoptotic damage to neurons by reducing the Bax/Bcl-2 ratio. In addition, scopolamine significantly decreased the expression level of BDNF and impaired the expression of neuroplasticity factors; however, WPH treatment (100 and 200 mg/kg) and co-treatment with WPH and treadmill exercise significantly increased BDNF expression (150.8%, 151.1%, 153.3%, and 168.3%, respectively; *p* < 0.05, Figure 4A,C). The combination of treadmill exercise and high-dose WPH administration synergistically increased BDNF protein levels.

Furthermore, WPH and treadmill exercise modulated the phosphorylation of tau proteins associated with neuronal function. Scopolamine administration significantly increased the p-tau/tau protein ratio, a marker of neurodegeneration, in the brain by 3.4-fold compared to that in the NOR group. However, the EXR, WPH_L, WPH_H, EWPH_L, and EWPH_H groups showed concentration-dependent reductions in the p-tau/tau ratio of 33.4%, 32.5%, 24.6%, 41.9%, and 28.9%, respectively (Figure 4A,D). These results indicate that treadmill exercise and WPH administration regulate neurogenesis and the neurodegenerative factors p-tau/tau and BDNF. 

### 3.5. Effects of Treadmill Exercise and WPH on Mitogen-Activated Protein Kinase (MAPK) Signaling in Scopolamine-Administered Mice 

**Figure 5:**
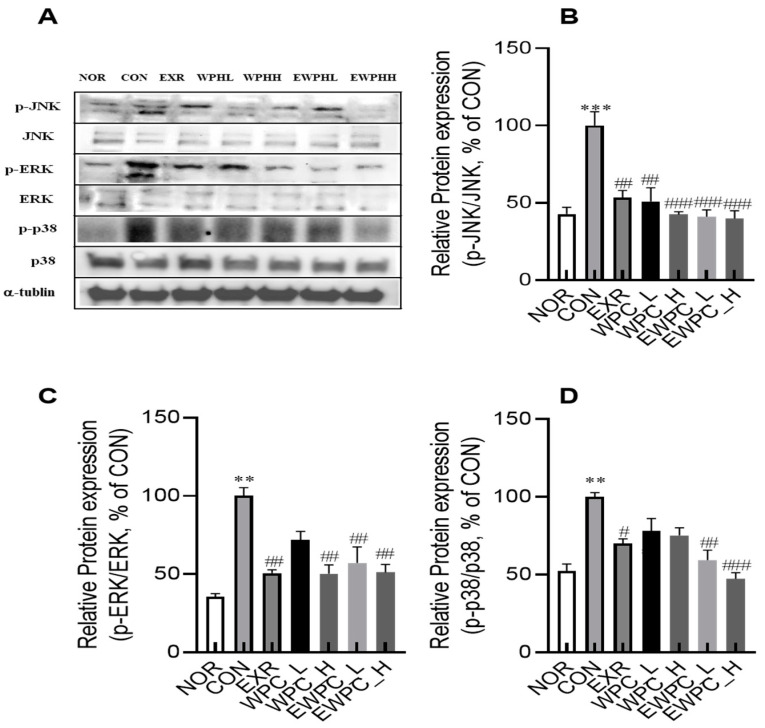
Effect of WPH and treadmill exercise on relative protein expression levels of the mitogen-activated protein kinase (MAPK) signaling pathway. NOR, normal; CON, control; EXR, exercise; WPH-L/H, low-dose/high-dose whey protein hydrolysate; EWPH_L/H, exercise + low-dose/high-dose whey protein hydrolysate. Western blot images of MAPKs (**A**). Relative protein levels of p-JNK/JNK (**B**), p-ERK/ERK (**C**), and p-p38/p38 (**D**) were determined using Western blot. Data are presented as mean ± standard error of the mean. ** *p* < 0.01, *** *p* < 0.001 vs. NOR group; ^#^ *p* < 0.05, ^##^ *p* < 0.01, ^###^ *p* < 0.001 vs. CON group (analysis of variance followed by post hoc Tukey’s test).

The effects of WPH and treadmill exercise on the MAPK signaling pathway were evaluated in the brain tissue. Scopolamine administration significantly increased the protein expression levels of p-c-Jun NH2-terminal kinase (JNK), p-extracellular signal-regulated kinase 1/2 (ERK1/2), and p-p38, key components of the MAPK signaling pathway, compared to those in the NOR group by 2.3-, 2.8-, and 1.9-fold, respectively (*p* < 0.01, Figure 5A–D). However, in the EXR, WPH_L, WPH_H, EWPH_L, and EWPH_H groups, the p-JNK expression was significantly reduced compared to that in the CON group by 53.3%, 50.9%, 42.4%, 41.1%, and 39.8%, respectively; p-ERK expression was reduced by 50.7%, 71.8%, 50.3%, 57.2%, and 51.1%, respectively; and p-P38 expression was reduced by 70.2%, 78.2%, 75.3%, 59.5%, and 47.3%, respectively (Figure 5B–D). These results indicate that exercise and WPH administration effectively inhibited the MAPK signaling pathway activated by scopolamine. Additionally, the inhibition of the MAPK signaling pathway was most pronounced when treadmill exercise and WPH administration were concomitantly performed.

### 3.6. Effects of Treadmill Exercise and WPH on Intestinal Microbiota

Cecal microbiota were measured to evaluate changes in the intestinal microbiota following WPH administration and treadmill exercise. The Shannon and Chao indices, which represent the diversity and abundance of the microbiome, respectively, tended to decrease in the CON group compared to those of the NOR group (Supplementary Figure S1). Treadmill exercise, WPH (100 and 200 mg/kg), and combined treatment with exercise and WPH (100 and 200 mg/kg) increased the Shannon index by 112%, 112%, 114%, 108%, and 110%, respectively, and the Chao index by 123%, 114%, 120%, 113%, and 116%, respectively, compared with those of the CON group (Supplementary Figure S1A,B). A principal coordinates analysis, which represents beta diversity, confirmed that the microbiome community changed between the NOR group and the CON group administered scopolamine. Treadmill exercise, WPH administration, and their combination resulted in a different microbiome community than that of the CON group (Supplementary Figure S1C). 

**Figure 6:**
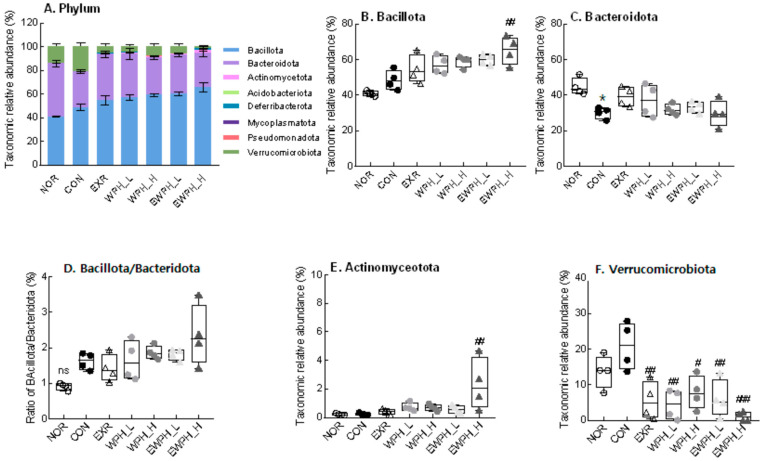
Effect of WPH and treadmill exercise on the gut microbiome composition at the phylum level. Taxonomic abundant compositions (**A**). The relative abundance of Bacillota (**B**), Bacteroidota (**C**), Bacillota/Baceroidota (**D**), Actinomyceotota (**E**), and Verrucomicrobiota (**F**). NOR, normal; CON, control; EXR, exercise; WPH-L/H, low-dose/high-dose whey protein hydrolysate; EWPH_L/H, exercise + low-dose/high-dose whey protein hydrolysate. The gut microbiome at the phylum level was analyzed using rRNA gene sequencing. Data are presented as mean ± standard error of the mean. * *p* < 0.05 vs. NOR group; ^#^ *p* < 0.05, ^##^ *p* < 0.01, ^###^ *p* < 0.001 vs. CON group; ns, non-significant (*p* > 0.05) (analysis of variance followed by post hoc Tukey’s test).

Changes in the cecal microbiota at the phylum level showed that treadmill exercise (EXR), WPH administration (WPH_L and WPH_H), and combined exercise and WPH administration (EWPH_L and EWPH_H) significantly reduced the relative abundance of *Verrucomicrobiota* that was increased in the CON group following scopolamine administration (Figure 6A). The exercise and high-dose WPH group (EWPH_H) had a significantly higher relative abundance of *Bacillota* than that of the CON group (Figure 6B). The *Bacillota/Bacteroidota* ratio increased after scopolamine administration. At the phylum level, *Bacillota* and *Bacteroidota* were the main taxa with high relative abundances, and there was no significant difference in their abundance among the groups (Figure 6B–D). The relative abundance of *Actinomycetota* in the EWPH_H group was significantly higher than that in the CON group (*p* < 0.01; Figure 6E), but did not differ significantly from that of the other groups. The combination of treadmill exercise and the high-dose administration of WPH showed a synergistic effect in increasing the relative abundances of *Bacillota* (Figure 6B) and *Actinomycetota* (Figure 6E) and in decreasing the relative abundance of *Verrucomicrobiota* (Figure 6F).

At the class and order levels, *Verrucomicrobiae* and *Verrucomicrobiales* had significantly decreased relative abundances in the EXR, WPH_L, WPH_H, EWPH_L, and EWPH_H groups compared to those of the CON group (*p* < 0.05, Supplementary Figure S2). The relative abundances of *Bacteroidia* and *Bacteroidales* were significantly higher in the NOR group than in the scopolamine-treated CON group. Furthermore, the relative abundance of *Actinomycetes* in the EWPH_H group was significantly higher than that in the CON group. 

Changes in the cecal microbiota were also detected at the family level. The relative abundance of *Akkermansiaceae* was significantly lower in the EXR, WPH_L, WPH_H, EWPH_L, and EWPH_H groups than in the CON group (*p* < 0.05, Supplementary Figure S2). The relative abundances of *Rikenellaceae* and *Clostridiaceae* were significantly higher in the WPH_H group than in the CON group (*p* < 0.05; Supplementary Figure S2). In addition, the relative abundance of *Eubacteriaceae* was significantly higher in the WPH_L and EWPH_H groups than in the CON group, whereas the relative abundance of *Lachnospiraceae* was significantly higher in the EXR group than in the CON group (*p* < 0.05, Supplementary Figure S2).

**Figure 7:**
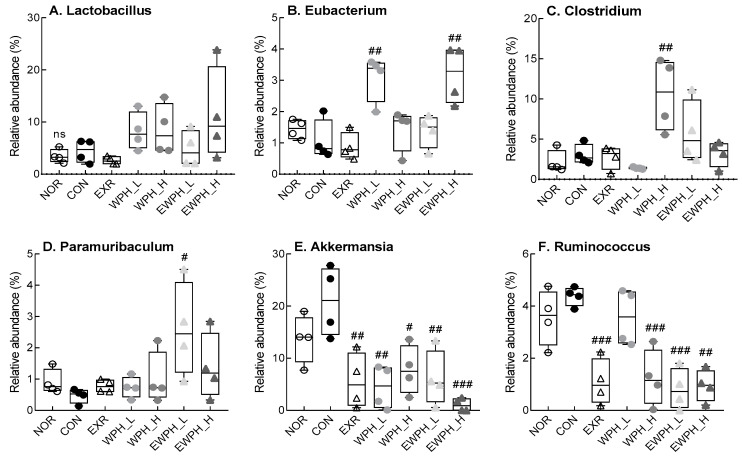
Effect of WPH and treadmill exercise on gut microbiome composition at the genus level. The relative abundance of Lactobacillus (**A**), Eubacterium (**B**), Clostridium (**C**), Paramuribaculum (**D**), Akkermansia (**E**), and Ruminococcus (**F**). NOR, normal; CON, control; EXR, exercise; WPH-L/H, low-dose/high-dose whey protein hydrolysate; EWPH_L/H, exercise + low-dose/high-dose whey protein hydrolysate. The gut microbiome at the genus level was analyzed using rRNA gene sequencing. Data are presented as mean ± standard error of the mean. ns; not significant. ^#^ *p* < 0.05, ^##^ *p* < 0.01, ^###^ *p* < 0.001 vs. CON group; ns, non-significant (*p* > 0.05) (analysis of variance followed by post hoc Tukey’s test).

At the genus level, WPH administration alone and in combination with treadmill exercise tended to increase the relative abundance of *Lactobacillus* compared to that of the CON group, but the difference was not statistically significant (Figure 7). The relative abundance of *Eubacterium* in the WPH_L and EWPH_H groups was significantly higher than that in the CON group (*p* < 0.01; Figure 7). Furthermore, the relative abundance of *Clostridium* was significantly increased in the WPH_H group compared to that of the CON group (*p* < 0.01, Figure 7). The relative abundance of *Paramuribaculum*, which decreased in the CON group, increased significantly in the EWPH_L group (*p* < 0.05; Figure 7). The relative abundances of *Akkermansia* and *Ruminococcus* were significantly lower in the EXR, WPH_L, WPH_H, EWPH_L, and EWPH_H groups than in the CON group. These results indicate that treadmill exercise and WPH protected against the scopolamine-induced alterations to the gut microbiota.

## 4. Discussion

In the present study, we investigated the effects of treadmill exercise and WPH intake on scopolamine-induced cognitive dysfunction in mice. Scopolamine, a muscarinic cholinergic receptor antagonist, induces cognitive impairment through cholinergic dysfunction and oxidative stress in the brain [21]. Therefore, animal models treated with scopolamine serve as reliable representations of cognitive impairment, and play a crucial role in investigations aimed at assessing the effectiveness of substances for the prevention and treatment of AD and uncovering their underlying mechanisms [22].

We found that WPH administration and treadmill exercise improved scopolamine-induced memory impairment in behavioral tests, including the Y-maze and novel object recognition tests. The Y-maze test is a straightforward method for assessing short-term spatial memory in experimental animals, which involves placing animals in a Y-maze and quantifying the number of sequential entries they make [23]. We found that scopolamine treatment significantly decreased spontaneous alternation behavior compared to that of the untreated NOR group. In addition, the EXR group, which received treadmill exercise; the WPH_L and WPH_H groups, which were supplemented with WPH; and the EWPH_L and EWPH_H groups, which received both exercise and WPH, showed a recovery of alternation behavior at levels similar to those in the NOR group, confirming that their memory was improved. The novel object recognition test has been widely used to study behavior and brain function in rats and mice, as well as in memory research. There was no significant difference in the time required to explore the novel and familiar objects after scopolamine administration [24]. However, the time spent exploring novel objects was significantly longer than the time spent exploring familiar objects in the EXR, WPH_L, WPH_H, EWPH_L, and EWPH_H groups. This suggests that exercise and WPH administration mitigate scopolamine-induced cognitive impairment. In our previous work (revision in progress), WPH has demonstrated its ability to protect neuronal cells from oxidative stress conditions, a finding that aligns with the current study’s data showcasing improved cognitive function. 

LDIQK, a WPH-derived pentapeptide (leucine–aspartate–isoleucine–glutamine–lysine), has been isolated using column chromatography and tandem spectrometry analysis and confirmed as an active principle for neuroprotective effects through its regulatory effect of calcium influx and the BAX/BCL2 ratio from our previous work [25]. However, the effect of LDIQK in animal models has not been described in this study. To confirm LDIQK as an active compound in WPH, purified LDIQK or LDIQK-rich fractions may be applied to animal models in further study. The present study substantiates the positive impact of WPH containing LDIQK on cognitive function in an animal model and introduces an additional intervention: exercise. This dual approach is believed to create a partial synergistic effect, further enhancing its ability. Exercise, a well-established contributor to overall health, is known to deliver a fresh supply of oxygen and nutrients to the brain by boosting blood flow. This physiological boost acts as a power-up for cognitive function, supporting optimal brain performance [26]. Furthermore, exercise has been reported to promote the release of neurotransmitters such as dopamine and serotonin, influencing mood and cognitive processes [27]. By comparing the neuroprotective effects of WPH with the cognitive benefits of exercise, our study suggests a partial synergistic relationship that could lead to a more significant improvement in cognitive ability. This dual intervention approach can provide a potential strategy for enhancing brain health and function.

The cholinergic nervous system plays a major role in cognition and memory, and scopolamine has been shown to increase AChE levels and decrease ACh and ChAT levels [20]. ACh is a major neurotransmitter and regulator in the nervous system, which is considered to play an important role in cognitive functions such as learning and memory at the neuromuscular junction and in the parasympathetic nervous system [28]. An increased activity of enzymes such as AChE and butyrylcholinesterase in the brain leads to cholinergic dysfunction by breaking down the neurotransmitter ACh into choline and acetyl-coenzyme A, which contributes to impaired memory and cognitive function [29]. In this study, we found that treadmill exercise and WPH administration significantly decreased the activity of AChE and increased the protein expression of ChAT, leading to an increase in the ACh concentration. These effects of exercise and WPH administration on ACh, AChE, and ChAT correlated with the results of the behavioral tests (Y-maze and novel object recognition), showing an increase in spontaneous alternation and the novel object discrimination index.

Along with its involvement in cognitive function, ACh is closely associated with oxidative stress in the brain. Given the abundance of unsaturated fatty acids in the brain tissue and their susceptibility to oxidative stress, the excessive production of free radicals leads to the accumulation of lipid peroxides, protein denaturation, DNA oxidation, and subsequent cell damage, thereby hindering physiological activities [29]. In particular, oxidative stress leads to the peroxidation of unsaturated fatty acids surrounding nerve cells and an increase in the activity of AChE, which promotes the degradation of ACh, resulting in impaired neurotransmission and, ultimately, a decrease in memory and learning ability. The findings of this study indicated that both exercise and WPH administration significantly reduced scopolamine-induced ROS and MDA levels. These results suggest that treadmill exercise and WPH improve cognitive function by inhibiting ACh degradation through a reduction in oxidative stress. 

Antioxidants can play a crucial role in protecting the brain neuronal cells and lowering the risk of cognitive decline by neutralizing free radicals. Some studies suggest that antioxidants or an antioxidant-rich diet may contribute to the improvement of cognitive function or neurodegenerative diseases [30,31,32]. Bonyadi et al. reported that berry-based foods have beneficial effects on cognitive function and memory performance [31]. A recent study showed the Mediterranean-DASH Intervention for Neurodegenerative Delay (MIND) diet may be a useful option for protecting various dementia pathologies [32]. 

The Bax/Bcl-2 ratio, which is increased by oxidative stress, is also an important factor in the regulation of apoptosis [33]. Bcl-2, Bcl-xL, and related factors inhibit apoptosis, whereas Bax, Blk, and Bad promote apoptosis. Bax translocates to the mitochondria, where it becomes an activated homodimer and promotes apoptosis; this Bax-induced cell death pathway is inhibited by its heterodimerization with Bcl-2. Previous studies linking exercise to synaptic plasticity and cell proliferation have shown that exercise increases the expression of Bcl-2, an anti-apoptotic marker, and proteins involved in synaptic efficacy and learning in key brain regions [34]. By contrast, exercise inhibits the expression of Bax, a member of the caspase family acting downstream of the proapoptotic pathway and a major promoter of cell death, thereby alleviating cognitive dysfunction. The results of this study demonstrated that treadmill exercise and WPH administration (100 and 200 mg/kg) significantly reduced the Bax/Bcl-2 ratio. These results imply that treadmill exercise and WPH inhibited apoptosis to exert positive effects on cognitive function.

One of the most prominent pathological features of AD is the accumulation of neurofibrillary tangles due to hyperphosphorylated tau protein in the brain, which is toxic to neurons [35]. Additionally, the deposition of tau protein leads to oxidative stress, resulting in extensive neuronal destruction [36,37,38]. The destruction of nerve cells in turn causes changes in the brain structure and function, leading to cognitive decline [36,37,38]. In particular, pronounced brain atrophy has been observed in the brains of patients with AD, and the administration of scopolamine has been reported to cause a decrease in brain weight along with a decline in overall cognitive function [39]. A recent study showed that whey protein administration reduced the high hyperphosphorylation levels of tau protein in aged rats [40], and another study demonstrated that treadmill exercise inhibited tau protein hyperphosphorylation in tau transgenic mouse models [41]. The results of the present study showed that four weeks of moderate-intensity treadmill exercise and WPH administration at concentrations of 100 and 200 mg/kg significantly inhibited tau protein hyperphosphorylation. The inhibition of p-tau through exercise and WPH administration may contribute to a reduction in neuronal apoptosis.

BDNF is a neurotrophic factor that regulates neuronal growth, increases the activity of acetylcholine synthase in the central nervous system, enhances synaptic plasticity, and is directly involved in memory storage and utilization [42,43,44]. Notably, transgenic mice with reduced BDNF expression showed impaired synaptic function, long-term memory, and learning, and BDNF expression in the brain was found to be reduced in patients with AD [45]. Consistently, in the present study, we found that BDNF protein expression was significantly reduced in the CON group following scopolamine administration. However, when WPH was administered in combination with treadmill exercise, BDNF expression significantly increased. This increase in BDNF levels after exercise and WPH administration may also be associated with a reduction in apoptosis and p-tau.

Under oxidative stress, MAPKs are signal transducers that regulate cell death [46]. The phosphorylation of MAPK proteins by scopolamine leads to an elevated Bax/Bcl-2 ratio [46]. The activation of the MAPK pathway (including ERK1/2, JNK, and p38 MAPK) is one of the markers observed in patients with AD [47]. Moderate-intensity treadmill exercise and WPH administration significantly reduced MAPK activation in scopolamine-induced mice. These results suggest that whey protein supplementation and treadmill exercise are effective in improving cognitive function by inhibiting the scopolamine-induced activation of MAPKs.

Food ingredients can enhance cognitive function by influencing the gut bacterial diversity. The gut microbiome, constituting 95% of the human microbiome, plays a vital role in the gut–brain axis [48]. This axis links the gut microbiome to the neurological, immune, endocrine, and metabolic systems of the host, thereby playing a role in various neurological and cognitive disorders [49]. *Actinomycetes* are significant contributors to these pathologies by inhibiting the activity of AChE, which breaks down ACh [50]. We found that high doses of WPH and treadmill exercise increased the relative abundance of *Actinomycetes* and reduced AChE activity. WPH administration and treadmill exercise also decreased and increased the relative abundance of *Verrucomicrobiota* and *Lachnospiraceae*, respectively. The relative abundance of *Lachnospiraceae* has been reported to be higher in healthy populations than in patients with AD [51].

Treadmill exercise, WPH administration, and their combined treatment decreased the relative abundances of *Ruminococcus* and *Akkermansia* at the genus level. A decrease in *Ruminococcus* abundance was also observed in the normal group during a clinical trial investigating the interaction between cognitive function and the gut flora, whereas an increase in *Ruminococcus gnavus* relative abundance was reported in a scopolamine-treated model of cognitive dysfunction [52]. These results suggest that cognitive dysfunction may be ameliorated through interactions with other microbes.

Changes in the relative abundance of *Akkermansia*, a bacterial genus belonging to the phylum *Verrucomicrobiota*, were similar to those of *Verrucomicrobiota* detected at the phylum level. *Akkermansia* was previously shown to ameliorate impaired glucose, fat metabolism, and intestinal epithelial cell damage in AD models [53]. However, an excessive increase in *Akkermansia muciniphila* did not effectively enhance memory in cognitively impaired mice. The relative abundance of *Clostridium* tended to increase in all groups in this study, except for the WPH_L group, and the relative abundance increased with higher concentrations of WPH administered. An increase in *Clostridium* was associated with enhanced synthesis of 3-indolepropionic acid, which scavenges free radicals, reduces neuronal cell death, and alters blood–brain barrier permeability to improve cognitive function [54]. These results suggest that treadmill exercise and WPH improve cognitive function by modulating the gut microbiota associated with cognitive function.

## 5. Conclusions

This study demonstrates that WPH intake and treadmill exercise prevent scopolamine-induced cognitive decline. WPH and treadmill exercise improved cognitive function by reducing ROS and MDA levels in the brain tissue and by increasing the concentration of ACh. WPH and exercise also protected neurons by inhibiting the hyperphosphorylation of tau proteins, increasing the expression of BDNF, and inhibiting apoptosis by downregulating the MAPK pathway. Furthermore, we found that WPH and treadmill exercise improved cognitive function by altering the composition of the gut microbiome, with an increase in the Chao and Shannon indices, suggesting that treadmill exercise and WPH are effective in ameliorating cognitive impairment, providing a potential strategy for addressing neurodegenerative diseases.

## Data Availability

The data presented in this study are available on request from the corresponding author. The data are not publicly available due to privacy.

## Supplementary Materials

The following supporting information can be downloaded at https://www.mdpi.com/article/10.3390/foods12244428/s1: Figure S1: Effects of WPH and treadmill exercise on gut microbiome diversity; Figure S2: Effect of WPH and treadmill exercise on gut microbiome composition at the class, order, and family levels.
