# Supplementary material for: Protective Effects of Whey Protein Hydrolysate, Treadmill Exercise, and Their Combination against Scopolamine-Induced Cognitive Deficit in Mice
Source: Foods. 2023 Dec 10;12(24):4428. doi: 10.3390/foods12244428 (PMC10742977; doi:10.3390/foods12244428)
Supplement: Supplementary file 1 [file foods-12-04428-s001.zip › foods-2742305-supplementary.pdf]

Supplementary Material

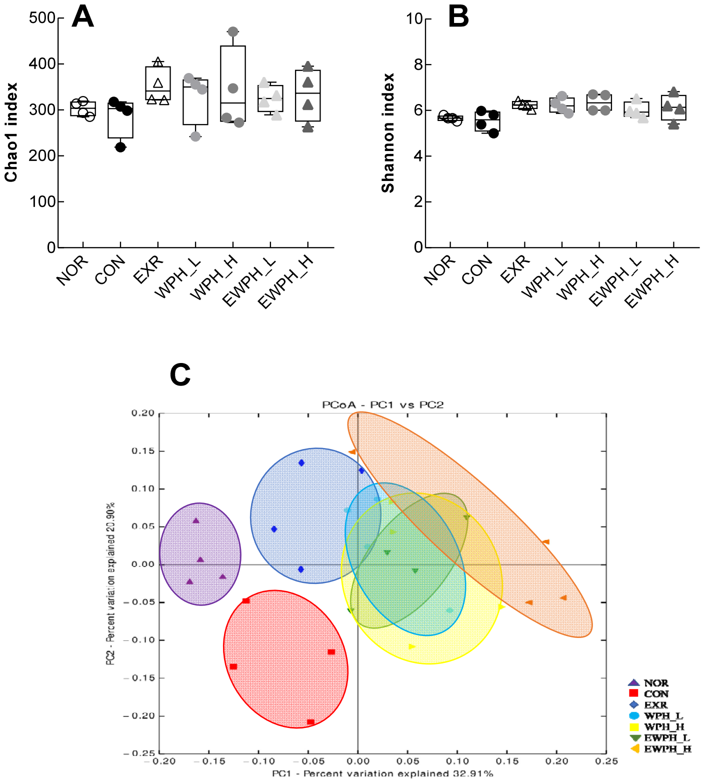

Supplementary Figure S1. (A–C) Effect of WPH and treadmill exercise on gut microbiome diversity.NOR, normal; CON, control; EXR, exercise; WPH-L/H, low-dose/high-dose whey protein hydrolysate; EWPH_L/H, exercise + low-dose/high-dose whey protein hydrolysate

A. Class & Order

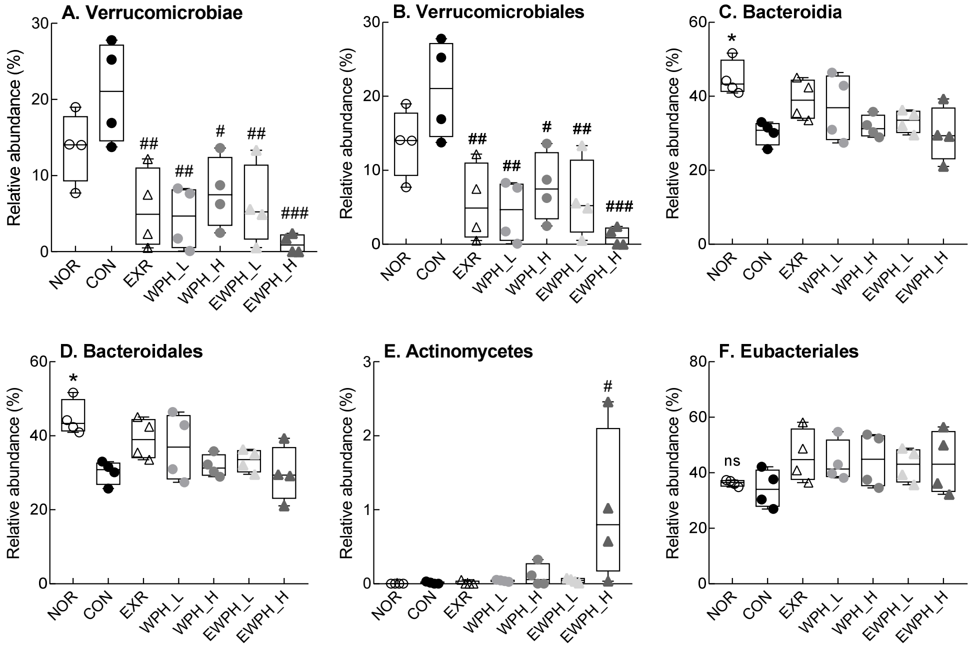

B. Family

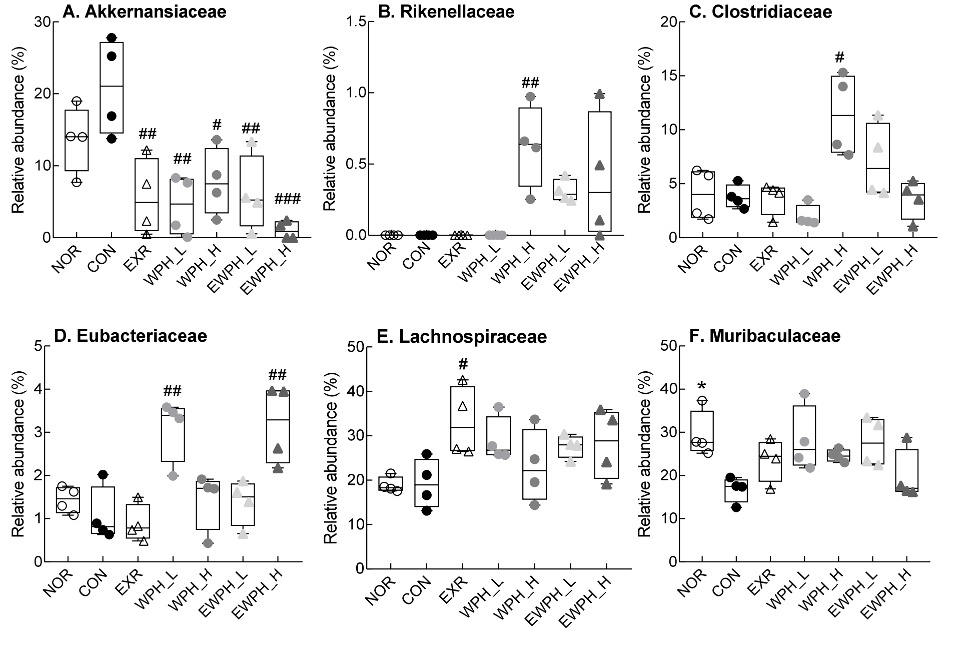

Supplementary Figure S2. Effect of WPH and treadmill exercise on the gut microbiome composition at the (A) class & order and (B) family level. NOR, normal; CON, control; EXR, exercise; WPH-L/H, low-dose/high-dose whey protein hydrolysate; EWPH_L/H, exercise + low-dose/high-dose whey protein hydrolysate.
